# Successful Treatment of Post-coronavirus Disease 2019 (COVID-19) Autoimmune Encephalitis With Plasmapheresis After a Failed Trial of Steroids

**DOI:** 10.7759/cureus.85150

**Published:** 2025-05-31

**Authors:** Tony Kamel, William Toppen, Yasaman Salahmand

**Affiliations:** 1 Department of Internal Medicine, David Geffen School of Medicine, University of California, Los Angeles (UCLA), Los Angeles, USA; 2 Department of Internal Medicine, University of California San Diego (UCSD) Health, San Diego, USA

**Keywords:** covid-19, intravenous steroids, plasmapheresis, post-viral encephalitis, rare cause of altered mental status

## Abstract

In December 2021, a 58-year-old unvaccinated woman with a past medical history of obesity presented to an outside hospital with severe coronavirus disease 2019 (COVID-19) pneumonia requiring intubation and a prolonged ICU stay. Although she eventually recovered and was extubated, she continued to experience persistent generalized weakness, nausea, weight loss, and mental status changes following discharge. In March 2022, she required re-hospitalization for altered mental status. Her condition progressively deteriorated, rendering her obtunded and nonverbal, responding only to noxious stimuli. After an extensive but unremarkable infectious, neurological, and hematologic workup, she was transferred to our facility for a higher level of care. Post-COVID-19 autoimmune encephalitis was suspected. Despite a trial of intravenous steroids, she remained largely nonverbal, producing only occasional single-word utterances. Following treatment with plasmapheresis, she demonstrated remarkable improvement, speaking in near-complete sentences just one day later and gradually recovering thereafter. It had previously been unclear whether plasmapheresis could serve as an effective treatment for post-COVID-19 encephalitis. We present a case of severe encephalopathy due to post-COVID-19 encephalitis that was successfully treated with plasmapheresis after failure of steroid therapy.

## Introduction

Coronavirus disease 2019 (COVID-19) can cause a myriad of symptoms affecting multiple organ systems, most notably the respiratory system; however, it may also lead to significant neurological complications. Central nervous system involvement can range from mild symptoms such as headaches, dizziness, and "brain fog" to more severe manifestations including altered mental status, seizures, cerebrovascular events, encephalitis, and encephalopathy. COVID-19 can also impact the peripheral nervous system, resulting in paresthesias and dysesthesias [1,2]. The pathophysiology underlying these neurological effects, as well as the variability in symptom presentation among individuals, remains incompletely understood [3].

Post-infectious complications of COVID-19 have been increasingly recognized and include persistent "brain fog" (cognitive dysfunction such as forgetfulness and difficulty thinking clearly), post-COVID-19 encephalitis, and, in some patients, "long COVID" which refers to symptoms persisting more than four weeks after primary infection [2]. More recently, data have emerged suggesting that some cases of post-COVID-19 encephalitis may respond to immunomodulatory therapies such as corticosteroids or intravenous immunoglobulin (IVIG), evidence that was not widely available during the early stages of the pandemic, including at the time of our patient's presentation [1-4]. While the role of plasmapheresis in treating post-viral encephalitis has been described in the context of non-COVID-19 infections, its use in COVID-19-related sequelae remains less well established [4,5] Here, we present a case of severe encephalopathy secondary to post-COVID-19 encephalitis in an unvaccinated patient, successfully treated with plasmapheresis following failure of corticosteroid therapy.

## Case presentation

A 58-year-old unvaccinated woman with a past medical history of obesity presented to an outside hospital in late December 2021 with severe COVID-19 pneumonia. She required intubation and mechanical ventilation and had a prolonged hospital stay. Although she eventually improved and was discharged, she subsequently experienced persistent nausea, daily emesis, abdominal pain, more than 100 pounds of unintentional weight loss, as well as progressive mental status changes. An extensive outpatient workup with her gastroenterologist, including esophagogastroduodenoscopy (EGD) and magnetic resonance cholangiopancreatography (MRCP), did not reveal a clear etiology. Due to ongoing symptoms, progressive generalized weakness, and progressive mental status changes, she presented to another outside hospital, where she was admitted for a three-week stay marked by worsening mental status, eventual obtundation, and recurrent fevers of unclear origin. She was evaluated by infectious disease and neurology teams, who directed her initial workup.

Laboratory studies were notable for neutrophil-predominant leukocytosis, lactic acidosis (which resolved with fluid resuscitation), and thrombocytosis. Lumbar puncture revealed a normal cerebrospinal fluid (CSF) white blood cell count (3 cells/mm^3^) and mildly elevated protein (68 mg/dL); cultures were negative. Inflammatory markers were elevated. Extensive brain imaging, including computed tomography (CT) and magnetic resonance imaging (MRI), showed no acute abnormalities. Although no definitive cause of her encephalopathy was identified, autoimmune encephalitis (AIE) was suspected, and the patient was transferred to our facility for a higher level of care. Since her initial diagnosis two months before transfer, and repeat hospitalization three weeks prior to transfer, she had not received any specific therapies targeted towards AIE. 

Upon arrival, the patient was encephalopathic, nonverbal, and unable to provide history or follow commands. She was frequently awake but did not track purposefully with her eyes and occasionally mumbled incoherently. Physical examination revealed a systolic murmur but was otherwise unremarkable.

**Figure 1:**
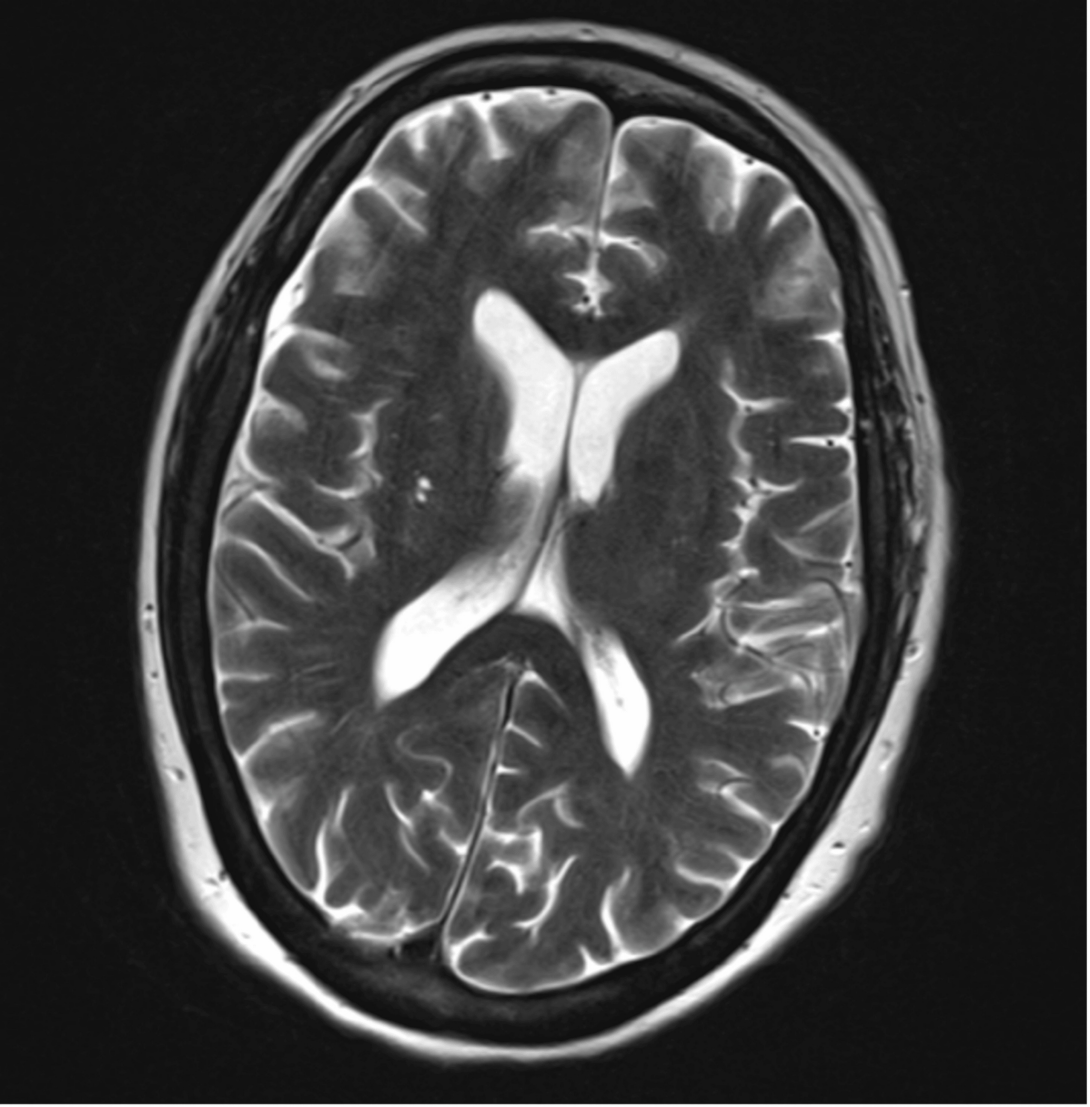
Brain MRI of the patient one day after admission MRI: magnetic resonance imaging

Infectious disease, neurology, and rheumatology teams were consulted and pursued an extensive diagnostic workup, which remained unrevealing. A repeat lumbar puncture demonstrated mild lymphocytic pleocytosis (9 cells/mm^3^) and mildly elevated protein (60 mg/dL) (Table 1). The electroencephalogram showed no seizure activity. A comprehensive meningoencephalitis panel was negative, and extensive testing for bacterial, viral, and parasitic infections was unrevealing (Table 2). Next-generation sequencing and Karius testing yielded no significant findings. Autoimmune antibody testing for antinuclear antibody (ANA), rheumatoid factor, Smith/ribonucleoprotein (Sm/RNP), and anti-Ro (SSA)/anti-La (SSB) were negative. Evaluation for nutritional deficiencies showed low vitamin D and folate levels (with otherwise normal vitamin B12, niacin, and thiamine), likely secondary to poor oral intake in the context of weight loss and persistent encephalopathy; both were corrected. Porphyria testing (porphobilinogen in urine and whole blood porphyrins) and Raji cell assay were normal. Repeat MRI of the brain with and without contrast, magnetic resonance angiography (MRA) of the head, and magnetic resonance venography (MRV) of the head were unremarkable (Figure 1). Given the exhaustive negative workup at both institutions and the absence of acute infection, post-infectious encephalitis secondary to her recent severe COVID-19 illness was presumed.

**Table 1:** CSF results from the outside hospital and repeat studies 10 days later upon admission WBC: white blood cell; RBC: red blood cell; CSF: cerebrospinal fluid; LP: lumbar puncture

	Initial LP	Repeat LP upon admission	Reference range
CSF WBC	3 mm^3^	9 mm^3^	0-5 mm^3^
CSF glucose	68 mg/dL	94 mg/dL	45-75 mg/dL
CSF protein	63 mg/dL	60 mg/dL	15-45 mg/dL
CSF RBC	1 mm^3^	8 mm^3^	0-10 mm^3^

**Table 2:** Comprehensive lab values including CBC, CMP, LFTs, and infectious disease tests of the patient one day after admission * indicates abnormal lab value CBC: complete blood count; CMP: comprehensive metabolic panel; LFTs: liver function tests; CMV: cytomegalovirus; PCR: polymerase chain reaction; CSF: cerebrospinal fluid; IgM: immunoglobulin M; IgG: immunoglobulin G; EBV: Epstein-Barr virus; RPR: rapid plasma reagin

	Value	Reference range
CBC		
Hemoglobin	12.8 g/dL	Female: 12.0-15.5 g/dL
		Male: 13.5-17.5 g/dL
Mean corpuscular volume	97.3 fL	80-100 fL
Platelet count	380×10³/µL	150-400×10³/µL
White blood cell count	17.76*×10³/µL	4.0-11.0×10³/µL
Neutrophil	77.2%	40-60%
Absolute neutrophil count	13.69*×10³/µL	1.5-8.0×10³/µL
Lymphocyte	10.8%	20-40%
Absolute eosinophil count	0.50×10³/µL	0.0-0.5×10³/µL
CMP		
Sodium	148* mmol/L	135-145 mmol/L
Potassium	3.6 mmol/L	3.5-5.0 mmol/L
CO2	20 mmol/L	22-29 mmol/L
Anion gap	13 mmol/L	8-16 mmol/L
Blood urea nitrogen	27* mg/dL	7-20 mg/dL
Creatinine	1.00 mg/dL	Female: 0.59-1.04 mg/dL
		Male: 0.74-1.35 mg/dL
LFTs		
Total protein	6.5 g/dL	6.0-8.3 g/dL
Albumin	3.6* g/dL	3.4-5.4 g/dL
Total bilirubin	1.1 mg/dL	0.1-1.2 mg/dL
Aspartate aminotransferase	47 U/L	10-40 U/L
Alanine aminotransferase	38 U/L	7-56 U/L
Alkaline phosphatase	107 U/L	40-129 U/L
Infectious disease tests		
CMV DNA qualitative PCR, CSF	Negative	Negative
Cocci antibodies IgM and IgG, complement fixation	Negative	Titer <1:2
Cryptococcal antigen CSF	Negative	Negative
EBV IgM	0 copies/PCR	0 copies/PCR
West Nile CSF IgG	0.18	IV <1.29
West Nile CSF IgM	0	IV <0.89
RPR	Nonreactive	Nonreactive
HIV 1 and 2 CSF PCR	Not detected	Undetectable
Meningitis/encephalitis PCR panel	Negative	Negative
Autoimmune encephalitis panel, CSF	Negative	Negative

After ruling out acute infectious causes of the patient's persistent encephalopathy, she was started on a five-day course of intravenous methylprednisolone 1 g daily beginning on hospital day 4 to treat suspected post-viral AIE. There was no significant change in her mental status following steroid treatment. On hospital day 17, she demonstrated minimal improvement, only occasionally saying the word "hello" but remaining largely non-communicative and unable to reliably follow commands. Due to her limited progress, the hematology/oncology team was consulted for the consideration of plasmapheresis based on existing literature, including a study by Dogan et al. [6]. She was treated with five days of plasmapheresis, and remarkably, by the following day, the patient was able to speak in nearly full sentences and follow commands, though she exhibited poor short-term memory. She continued to improve gradually and was discharged to a skilled nursing facility on hospital day 45. Her speech and cognitive dysfunction have continued to recover in the outpatient setting, and she has not required re-hospitalization since.

## Discussion

COVID-19 is well known for its respiratory manifestations but may also result in serious neurological complications, including post-viral AIE, as seen in our patient. While the treatment of post-viral AIE from other viral pathogens has been more thoroughly studied, the efficacy of these treatments for COVID-19-related encephalitis remains less well established. Our case highlights plasmapheresis as a potentially effective therapeutic option for post-COVID-19 AIE, particularly in patients who do not respond to corticosteroids [4,5].

After failing a trial of intravenous steroids, our patient was treated with plasmapheresis based on the case series reported by Dogan et al., in which six patients with COVID-19-associated autoimmune meningoencephalitis demonstrated clinical improvement following plasmapheresis [6]. The proposed mechanism of action involves the removal of pathogenic autoantibodies and proinflammatory cytokines from the circulation, thereby restoring immunologic homeostasis [7-9]. Although the specific immunopathology of COVID-19-related AIE is still being elucidated, it is reasonable to infer that the mechanism of plasmapheresis parallels to that observed in other forms of post-viral AIE [6-8].

Considering our patient's significant and rapid improvement following plasmapheresis after failing steroid therapy, we propose that plasmapheresis may represent a valuable treatment for post-COVID-19 AIE.

## Conclusions

Plasmapheresis may be a viable treatment modality for post-COVID-19 AIE, particularly in patients who do not respond to corticosteroids. This case highlights a rare but serious complication of COVID-19, in which a previously functional patient developed profound encephalopathy months after recovering from acute infection. Despite an extensive diagnostic workup and initial treatment with intravenous steroids, the patient's condition remained largely unchanged until she received plasmapheresis, which resulted in a rapid and significant improvement in mental status. Given the growing recognition of post-infectious neurologic syndromes associated with severe acute respiratory syndrome coronavirus 2 (SARS-CoV-2), this case contributes to the emerging evidence suggesting that plasmapheresis may play a role in the treatment algorithm for autoimmune neurologic complications of COVID-19. Further research is needed to better understand the pathophysiology of post-COVID-19 encephalitis and to define optimal treatment strategies, but clinicians should consider plasmapheresis, especially if other immunosuppressive therapies fail.
